# Address of President Andrews

**Published:** 1888-10

**Authors:** 


### PRESIDENT ANDREWS' ADDRESS TO THE UNION MEETING.

*Ladies and Gentlemen; Guests of the Union Meeting of Dentists:*—It is an honorable privilege to welcome you one and all to the hospitalities and fraternal courtesies of our city, and, in doing so, I may be pardoned if I acknowledge some little pride in being the chosen representative of so large a constituency of my brethren of the dental profession. I welcome you, not alone in the name of the Connecticut Valley Society; but the Massachusetts Dental Society also joins most heartily with us, in extending to you all a most cordial invitation to share with us the pleasures which have been provided for your entertainment. To our brothers from Canada, I would say, we have not forgotten the delightful memories of a year ago. In the growing interest manifested in our noble profession, you were foremost in extending to us, your brothers of this free Republic, the cordial welcome of your royal hearts. We have learned from you that fraternity knows no barriers of political difference; but that in the pursuit of knowledge, and in the development of professional skill, the true brotherhood of rights belong to all who are faithful in the discharge of honorable duty. I therefore feel a peculiar pride in giving you welcome to this old historic city of Boston, the cradle of our liberties, and the traditional mother of most of our intellectual activities. I recognize with grateful memory that the blood of our common heritage is the noble blood of England. We are blood relations by our common ancestry, and, in this kinship, I bid you, as brothers, to share our pleasures. We shall not try to vie with you in the largeness of our scheme of entertainment, for we have been so well warned, by our experiences with you, that if we had fostered the spirit of emulation in this attempt to give you pleasure, we might have hesitated with wisdom for our teacher. We shall never forget your lovely city and its many places of public interest; our delightful rides through your rich valleys, and over your noble rivers; nor the *grand* old pinnacle of Mount Royal, where beneath our feet we saw your splendid country spread out like a land of promise. We cannot forget your untiring and painstaking energy to open every avenue of enjoyment to our willing eyes; and now, that you are our guests, we want you to thoroughly rest, and let us do the serving. We throw open to you the doors of every place of interest, and bid you enter and enjoy yourselves. Old Boston has the reputation abroad of being a very crooked city. It matters a great deal to you, as it does to us, how that reputation is interpreted by the visitor. We make no boast of exceptional morality, but if by crookedness is meant successful uncertainty of street terminations, we have only to caution you the *straight and narrow way* is sometimes a fond delusion in Boston. But let me add, that to get lost anywhere in this town while you are in our hands will be a moral impossibility. We know that you will respond cordially to the sentiment of the profession, and its earnest friends everywhere, that dentists, as a class, are always safe in council, as they are in practice, and that wherever progress means enlightenment and scientific advancement, the profession of dentistry with even and steady step marches in the front rank of honorable consideration. The citizens of this old Commonwealth will therefore feel a just pride with us, in having among them as guests, our brethren of Canada, as well as those from our own States, who have done so much towards maintaining the dignity, and elevating the practice of a profession that has been such a blessing to humanity. In the name of our brotherhood of dentists I extend to you again a cordial welcome, expressing at the same time my sanguine hope that this reunion will redound to a closer friendship, a warmer sympathy of professional interests, a deeper realization of our unity, and, above all, a steadier advancement toward the attainment of our highest ideals of professional success. May your visit be one of continual pleasure, and when you seek again the busy round of daily duty, among the brightest pictures of your imagination,

> "As dreary time,
> Leans on the hand of thought,"

May you, looking backwards, recall, with pleasant memories, your visit here.

